# Sulfur Supplementation Enhances Cadmium Tolerance in Rice by Modulating Reactive Oxygen Species Scavenging, Thiol-Dependent Detoxification, and Mineral Nutrient Homeostasis

**DOI:** 10.3390/antiox15040467

**Published:** 2026-04-09

**Authors:** Ha-il Jung, Chaw Su Lwin, Myung-Sook Kim, Eun-Jin Lee, Tae-Gu Lee, Theint Thandar Latt, Jinwook Lee, Bok-Rye Lee

**Affiliations:** 1 Soil and Water Environment Division, National Institute of Agricultural Sciences, Rural Development Administration, Wanju 55365, Republic of Korea; hj255@korea.kr (H.-i.J.); msk74@korea.kr (M.-S.K.); eunjin0219@korea.kr (E.-J.L.); leetg7942@korea.kr (T.-G.L.); 2 Department of Plant Science and Technology, Chung-Ang University, Anseong 17546, Republic of Korea; theintthandarlatt@gmail.com (T.T.L.); JL425@cau.ac.kr (J.L.); 3 Institute of Environmentally-Friendly Agriculture (IEFA), Chonnam National University, Gwangju 61186, Republic of Korea

**Keywords:** cadmium, rice, sulfur, thiol-containing compounds, detoxification

## Abstract

Cadmium (Cd) is a potentially toxic element that impairs plant growth and threatens food safety and human health. This study aimed to investigate the effects of sulfur (S) supplementation on Cd uptake and tolerance in rice under hydroponic conditions. Rice seedlings were exposed to Cd stress and treated with S at different concentrations. Physiological traits, oxidative damage markers, thiol compounds, and ionomic profiles in rice plants were assessed. S supplementation reduced Cd-induced growth inhibition, restoring plant biomass. Although Cd accumulation increased with S treatment, it was accompanied by enhanced antioxidant responses, scavenging reactive oxygen species (ROS) and malondialdehyde. S application increased the production of thiol-containing compounds, including γ-glutamylcysteine, glutathione, and phytochelatins, which helped chelate Cd and sequester it in vacuoles, particularly in roots. Additionally, S supplementation altered the essential nutrient composition in rice tissues, particularly the uptake of N, P, and K, while influencing levels of Ca, Mg, and other essential elements. S supplementation enhanced rice tolerance to Cd stress by reestablishing ROS balance, activating thiol-based detoxification pathways, and regulating mineral nutrient balance. Furthermore, sulfur (S) exhibited a dual effect in plants, enhancing cadmium (Cd) uptake while also promoting its detoxification, underscoring its role in improving crop resilience in contaminated soils.

## 1. Introduction

Sulfur (S) is an essential macronutrient with diverse roles in plant physiology, particularly in amino acid biosynthesis, protein structure, and the formation of S-containing secondary metabolites involved in stress tolerance and metabolic regulation [1,2,3]. It is critical for maintaining cellular integrity and optimizing electron transport function while also supporting photosynthetic efficiency through its involvement in chlorophyll synthesis and nitrogen assimilation [4].

Under abiotic stress conditions, such as drought, salinity, extreme temperatures, and contamination by potentially toxic elements (PTEs), S metabolism becomes increasingly important. It promotes the activation of antioxidant defense systems and redox regulation by facilitating the biosynthesis of enzymatic antioxidants and thiol-containing compounds, including cysteine (Cys), methionine, γ-glutamylcysteine (γ-EC), glutathione (GSH), and phytochelatins (PCs) [2,5,6]. These metabolites are essential for PTE detoxification and help mitigate oxidative damage caused by reactive oxygen species (ROS).

Plants absorb S mainly as sulfate ions (SO_4_^2−^) from the rhizosphere through high-affinity sulfate transporters (SULTRs) [7,8,9,10], which mediate root uptake, xylem loading, and long-distance translocation to aboveground tissues [11]. Within plant cells, sulfate is reduced to sulfide and incorporated into Cys, a precursor for γ-EC and GSH synthesis. GSH serves as a major antioxidant and a precursor for PC production. These sulfhydryl functional groups facilitate chelation of toxic elements in the plant cells and then transfer and store them in vacuoles (e.g., via the formation of Cd–GSH and Cd–PC complexes), thereby alleviating their toxic effects on plants [12,13,14].

Cd is a non-essential, highly toxic element recognized as one of the most hazardous environmental pollutants due to its high mobility in soil and strong bioaccumulative properties. Its accumulation in edible rice grains raises serious food safety concerns. Chronic Cd exposure is associated with renal dysfunction, skeletal deformities, respiratory disorders, and elevated cancer risk [15,16]. S supplementation has thus emerged as a viable strategy for improving Cd tolerance in rice by boosting antioxidative responses, modulating S-dependent detoxification pathways, and restoring nutrient balance. However, the precise mechanisms by which sulfur mediates Cd stress mitigation are not yet fully understood and may vary with application dosage, chemical speciation, and dynamic soil–plant interactions.

This study aimed to investigate the effects of S application on the physiological and biochemical responses of rice plants under Cd stress. The specific objectives were to assess plant growth parameters, Cd accumulation profiles, essential ion homeostasis, oxidative stress markers, namely ROS and malondialdehyde (MDA), and the synthesis of S-containing thiol compounds, including γ-EC, GSH, and PCs. These assessments contribute to a deeper understanding of S-mediated Cd detoxification mechanisms, thereby providing sustainable agronomic strategies for rice cultivation in Cd-contaminated environments.

## 2. Materials and Methods

### 2.1. Plant Growth Conditions and Experimental Design

Seeds of rice (*Oryza sativa* L. cv. Shindongjin) were surface-sterilized by immersion in 70% ethanol for 2 min, followed by five rinses with distilled water. They were then disinfected by soaking in 8% ipconazole, a commercial fungicide, for 48 h, thoroughly rinsed, and germinated in deionized water under soaked conditions at 30 °C for 48 h. Uniformly germinated seedlings were transferred to a hydroponic culture system and cultivated until the two-leaf stage. Subsequently, the seedlings were transplanted into plastic pots (30 cm × 25 cm × 15 cm; eight seedlings per pot; surface area, 0.075 m^2^) containing 9 L of hydroponic nutrient solution and maintained until the five-leaf stage. The standard nutrient solution contained 1 mM S, 1.5 mM NH_4_NO_3_, 0.505 mM KH_2_PO_4_, 0.045 mM K_2_HPO_4_, 4 mM KCl, 3 mM CaCl_2_, 1 mM MgCl_2_, 1 mM MgSO_4_, 0.4 mM Fe-EDTA, 14 μM H_3_BO_3_, 5 μM MnCl_2_, 0.7 μM CuCl_2_, 3 μM ZnCl_2_, and 0.7 μM (NH_4_)6Mo_7_O_24_. The pH was adjusted to 5.8, and the solution was changed every 5 days of culture [17,18]. S treatment levels were established by modifying the nutrient solution. For S-deficient conditions (0 mM S), 1 mM MgCl_2_ and 1 mM MgSO_4_ were substituted by 2 mM MgCl_2_. For S-excess conditions (4 mM S), 4 mM KCl, 1 mM MgCl_2_, and 1 mM MgSO_4_ were replaced by 2 mM K_2_SO_4_ and 2 mM MgSO_4_.

Seedlings were cultivated in a greenhouse at the National Institute of Agricultural Sciences, Rural Development Administration in Wanju, Republic of Korea, under natural sunlight, with day/night temperatures of 30/25 °C and relative humidity of 60%/80%. Pots were arranged in three biological replicates per treatment and repositioned daily to minimize environmental variability. After reaching the five-leaf stage, seedlings were exposed to 10 μM Cd (CdCl_2_) in combination with 0, 1, and 4 mM S for 15 days. At the end of the treatment period, fresh root and leaf tissues were harvested, flash-frozen in liquid nitrogen, and stored at −80 °C for subsequent analyses of ROS, thiol compounds, and PCs.

### 2.2. Plant Growth Responses

The effect of S on the growth characteristics of rice seedlings cultivated under Cd-treated hydroponic conditions was evaluated by measuring plant height, shoot dry weight (DW), and root DW. After 15 days of treatment, seedlings were separated into roots and shoots, oven-dried at 80 °C for 72 h, and subsequently weighed.

### 2.3. Quantitative Analysis of Cd and Mineral Nutrient Contents

Root and shoot tissues of rice seedlings were harvested, thoroughly rinsed with tap water, and subsequently washed five times with distilled water to remove surface residues. The cleaned samples were oven-dried at 80 °C for 72 h and ground into a fine powder using a stainless-steel grinder. For elemental analysis, 200 mg of dried powder from each sample was digested using a Graphite Block Acid Digestion System (ODLAB Co., Ltd., Seoul, Republic of Korea). After complete digestion, the samples were cooled to room temperature, diluted to 100 mL with ultrapure water, and filtered through Grade 40 filter paper (Whatman International Ltd., Buckinghamshire, UK). The filtrates were analyzed for cadmium (Cd) and mineral nutrients using inductively coupled plasma–mass spectrometry (ICP–MS; Agilent 7900, Agilent Technologies Inc., Santa Clara, CA, USA). The quantified elements included P, K, Ca, Mg, Si, Fe, Mn, Cu, Zn, B, and Mo. Additionally, N and S contents were determined using a CNS elemental analyzer (Vario MAX CNS, Elementar Analysensysteme GmbH, Langenselbold, Germany). Cd accumulation in rice was calculated as follows:

(1) Cd accumulation = Ca × Cb

where Ca is the Cd concentration, and Cb is the dry weight of the root or shoot.

### 2.4. Assessment of ROS Generation and Lipid Peroxidation

Superoxide anion radical content was determined spectrophotometrically according to a previously described method [19] using a standard calibration curve of NaNO_2_. H_2_O_2_ content was determined using a colorimetric assay as described by [20]; absorbance was recorded at 410 nm, and concentrations were calculated using an extinction coefficient of 0.28 µM^−1^·cm^−1^. Lipid peroxidation was indirectly assessed by measuring MDA levels using the thiobarbituric acid (TBA) method, as previously described [21], with minor modifications. Absorbance at 532 nm was measured and corrected for nonspecific turbidity by subtracting the absorbance at 600 nm. MDA concentrations were calculated using an extinction coefficient of 156 mM^−1^·cm^−1^.

### 2.5. Quantification of Intracellular S-Containing Peptides (γ-EC and GSH) and Metal Chelators (PCs)

S-containing peptides, including γ-EC and GSH, as well as PCs, were extracted from fresh root and shoot tissues according to previously described methods [22,23], with some modifications. For each sample, 250 mg of tissue was homogenized in 300 μL of extraction buffer containing 5% (*w*/*v*) 5-sulfosalicylic acid, 6.3 mM diethylenetriaminepentaacetic acid, and 2 mM tris(2-carboxyethyl)phosphine. The mixture was vortexed for 30 s and shaken using a Mixer Mill (MM 400, Retsch GmbH, Haan, Germany) at 30 s^−1^ for 5 min. Samples were then placed in an ice bath for 15 min to stabilize thiol compounds, followed by a second shaking cycle (30 s^−1^ for 5 min) to enhance protein unfolding and PC release. The homogenates were centrifuged at 13,000 rpm for 10 min at 4 °C, and the resulting supernatants were filtered through 0.22 μm membrane filters prior to analysis.

GSH, γ-EC, and PC species (PC2, PC3, and PC4) were quantified using liquid chromatography coupled with triple quadrupole mass spectrometry (Nexera X_2_ UHPLC, Shimadzu Corp., Kyoto, Japan) as previously described [24], with minor adjustments to optimize detection specificity for rice tissue matrices.

### 2.6. Multivariate Data Analysis and Visualization

Multivariate data analysis was conducted to examine the relationships among plant growth characteristics, ROS, thiol compounds, Cd concentrations, and mineral nutrient levels in the roots and shoots of rice seedlings under Cd stress and non-stress conditions at varying S levels (0, 1, and 4 mM). Heatmaps of Pearson’s correlation coefficient were generated using MetaboAnalyst v.6.0 (https://www.metaboanalyst.ca) [25]. Prior to analysis, all data were normalized by autoscaling. Both scores and loading plots from principal component analysis (PCA) were constructed in the online platform MetaboAnalyst to identify variables that differentiated the treatment groups. Additionally, pairwise correlation coefficient networks of all measured variables were constructed using the MetScape v.3.1.3 plugin in Cytoscape v.3.8.2, as previously described [26]. In these networks, nodes represented the measured variables, and edges indicated significant correlations, offering insights into the interconnected responses of the plant system to different S and Cd treatments.

### 2.7. Statistical Analysis

Analysis of variance (ANOVA) was conducted for all datasets, and significant differences among treatments were determined using Duncan’s multiple range test. Statistical analyses were performed using SAS v.9.2 (SAS Institute Inc., Cary, NC, USA). Differences were considered statistically significant at *p* < 0.05. Data presented in the figures are expressed as mean ± standard deviation.

## 3. Results

### 3.1. S Supplementation Mitigated Cd-Induced Growth Suppression in Rice Seedlings

**Figure 1:**
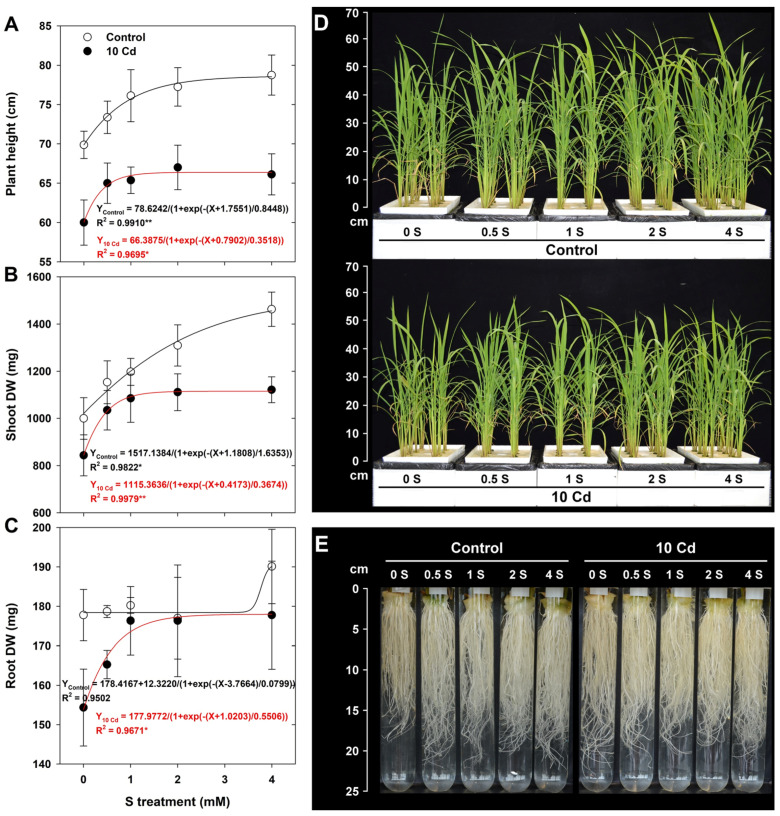
Effect of sulfur (S) on the growth characteristics of rice seedlings grown in cadmium (Cd)-treated hydroponics. (**A**) Plant height, (**B**) shoot dry weight (DW), and (**C**) root DW. Representative pictures of shoots (**D**) and roots (**E**) 15 days after treatment. The data represent the mean ± standard deviation of three or eight replicates. Asterisk indicates significant differences from control at * *p* < 0.05 and ** *p* < 0.01.

Cd stress significantly inhibited plant height, shoot DW, and root DW (Figure 1A–C) compared to those of the untreated control. However, S supplementation markedly improved all growth parameters, exhibiting a concentration-dependent recovery trend. In particular, treatments with 2 and 4 mM S led to the most pronounced enhancements in both shoot and root biomass under Cd exposure, suggesting an augmented tolerance mechanism. Visual phenotypic observations (Figure 1D,E) were consistent with the quantitative findings. Cd-treated plants without S supplementation displayed reduced growth and shortened root length. By contrast, seedlings receiving S exhibited restored shoot vigor and improved root architecture. These visual changes were consistent with the quantitative data. Collectively, these results highlight the alleviating role of S in mitigating Cd-induced growth inhibition in rice.

### 3.2. S Supplementation Influenced Cd Content and Accumulation in Rice Seedlings

**Figure 2:**
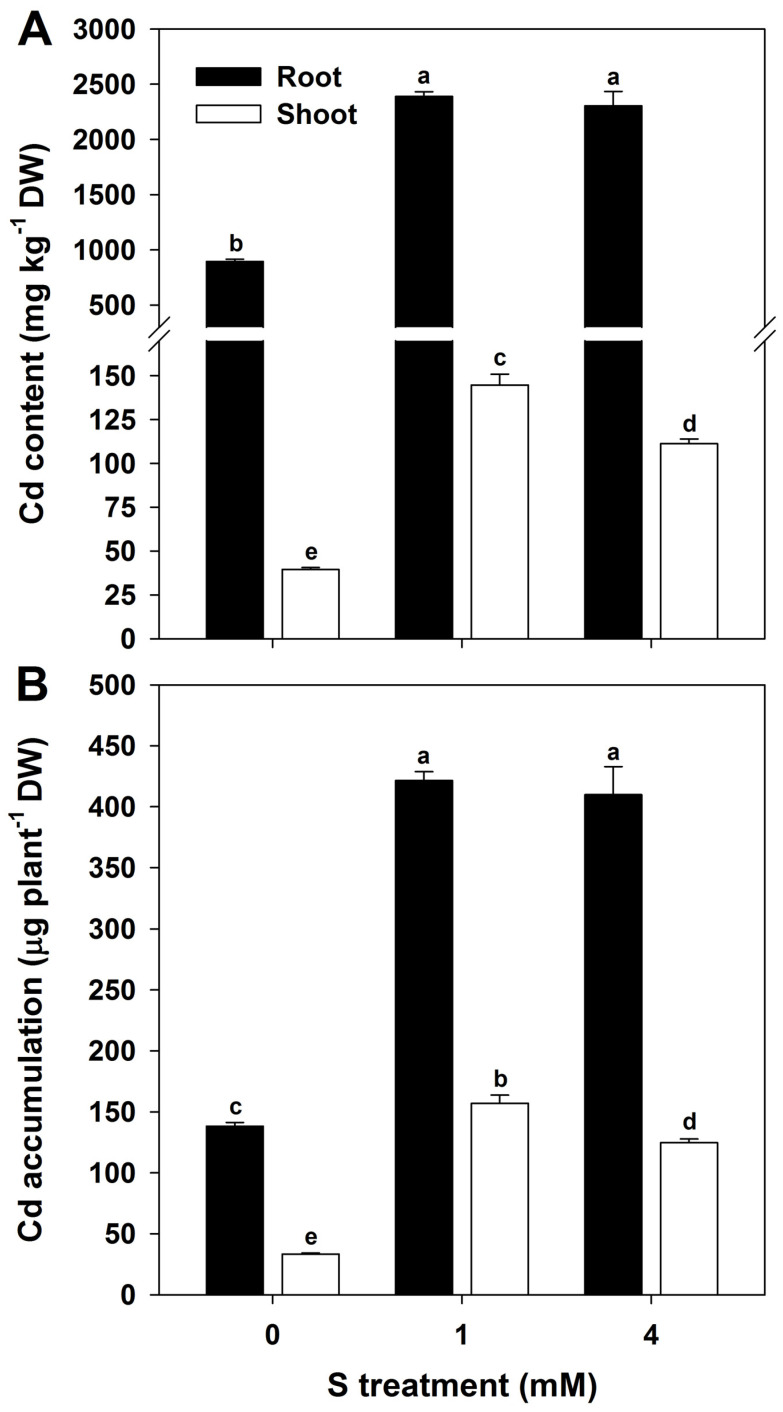
Effect of sulfur (S) on cadmium (Cd) contents (**A**) and Cd accumulation (**B**) in rice plants grown in Cd-treated hydroponics. The data represent the mean ± standard deviation of three replicates (*n* = 3). Means denoted by the same letters are not significantly different at *p* < 0.05.

S supplementation significantly increased Cd content in both root and shoot tissues of rice seedlings. In roots, Cd concentrations were markedly elevated following S treatment, with the highest levels observed at 1 and 4 mM concentrations. Shoots also exhibited an overall upward trend in Cd content, although slight variations were noted between S treatment levels (Figure 2A). Cd accumulation per plant closely followed the tissue-specific concentration patterns. Seedlings receiving S treatment showed significantly higher Cd accumulation in both roots and shoots than in the untreated control (Figure 2B).

### 3.3. S Supplementation Mitigated Cd-Induced Oxidative Stress in Rice Seedlings

**Figure 3:**
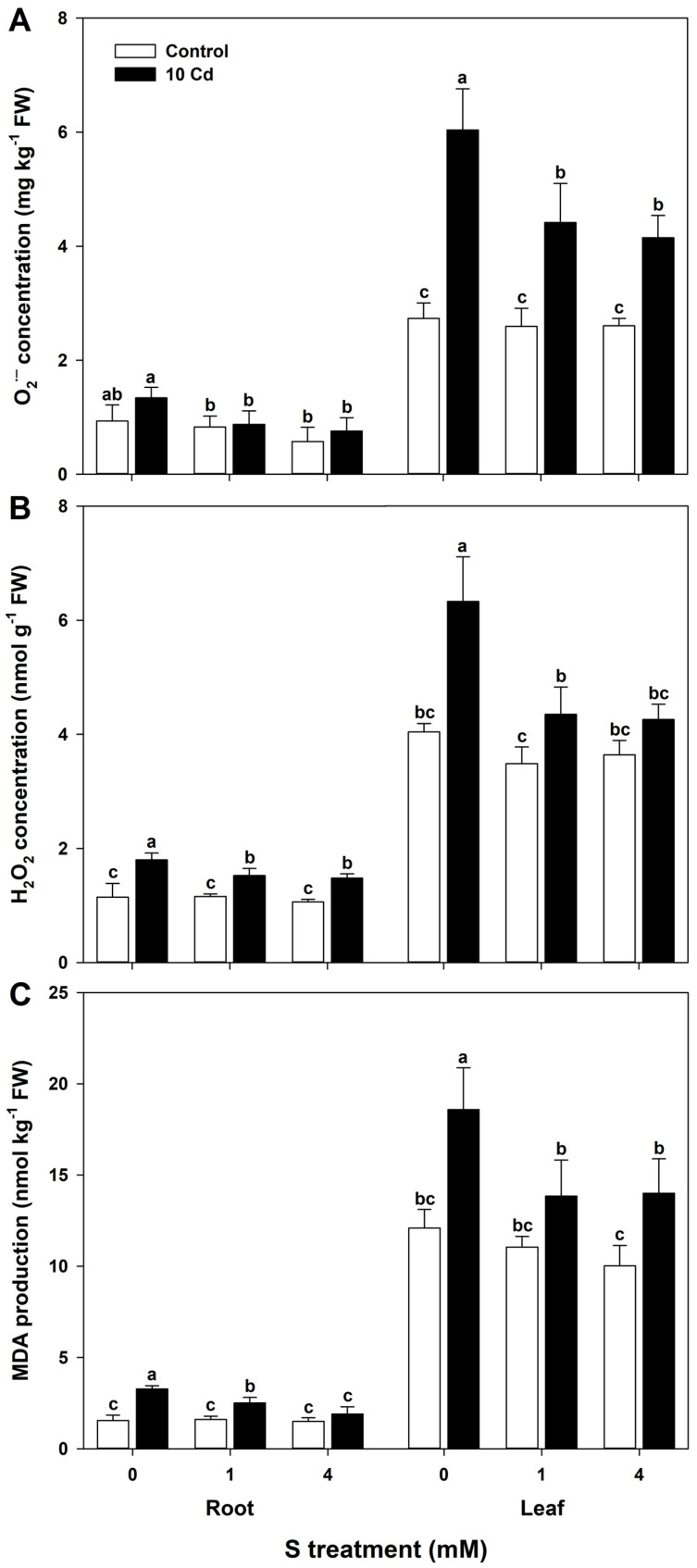
Effect of sulfur (S) on (**A**) superoxide anion radical (O_2_^•−^), (**B**) hydrogen peroxide (H_2_O_2_), and (**C**) malondialdehyde (MDA) levels in rice plants grown in cadmium (Cd)-treated hydroponics. The data represent the mean ± standard deviation of three replicates (*n* = 3). Means denoted by the same letters are not significantly different at *p* < 0.05.

Cd exposure markedly increased the accumulation of superoxide anion radical and H_2_O_2_ in the root and leaf tissues of rice seedlings, with the highest ROS levels observed in leaves without S supplementation (Figure 3A,B). S supplementation, particularly at 4 mM, significantly reduced ROS levels, indicating an improved antioxidative defense response (Figure 3A,B). Oxidative damage caused by Cd stress was further substantiated by elevated MDA levels, a reliable indicator of lipid peroxidation and membrane damage. Leaf tissues without S supplementation exhibited the highest MDA accumulation, whereas S-treated plants showed a substantial reduction in MDA content, suggesting enhanced membrane integrity and mitigation of oxidative stress (Figure 3C).

### 3.4. S Supplementation Enhanced Thiol Metabolism in Rice Seedlings Under Cd Stress

**Figure 4:**
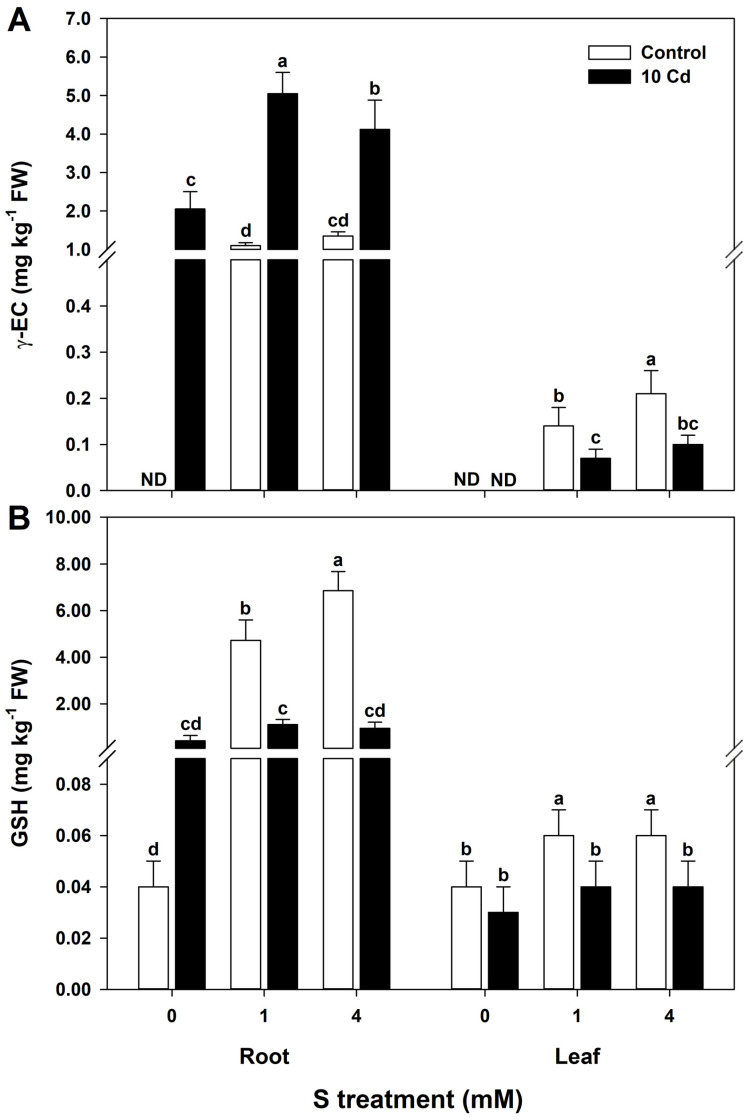
Effect of sulfur (S) on (**A**) gamma-glutamylcysteine (γ-EC) and (**B**) glutathione (GSH) contents in rice plants grown in cadmium (Cd)-treated hydroponics. The data represent the mean ± standard deviation of three replicates (*n* = 3). Means denoted by the same letters are not significantly different at *p* < 0.05. ND, not detected.

Cd exposure significantly enhanced γ-EC accumulation in the root tissues of rice seedlings, with the highest concentration observed under 1 mM S treatment. A moderate increase was also detected in 4 mM S-treated plants, while untreated control plants maintained low γ-EC levels. In leaf tissues, γ-EC content peaked in seedlings supplemented with 4 mM S but declined sharply under Cd stress, regardless of S supplementation (Figure 4A). GSH levels followed a similar trend. The highest GSH concentrations were detected in both roots and leaves of rice seedlings supplemented with 1 mM S. However, under Cd stress, GSH content decreased significantly across all treatments, indicating possible thiol depletion under prolonged stress conditions (Figure 4B).

### 3.5. S Supplementation Promoted PC Biosynthesis in Rice Seedlings Exposed to Cd

**Figure 5:**
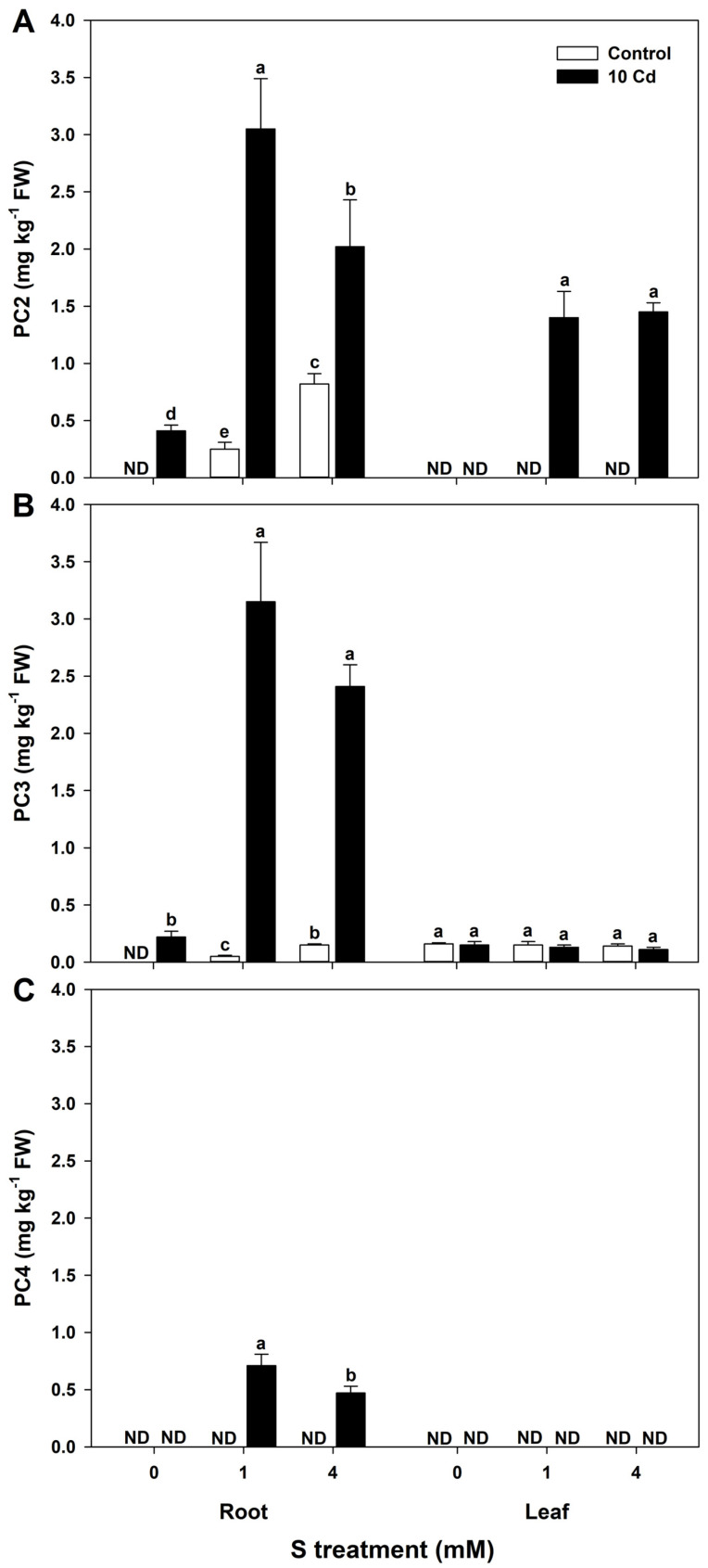
Effect of sulfur (S) on (**A**) PC2, (**B**) PC3, and (**C**) PC4 contents in rice plants grown in cadmium (Cd)-treated hydroponics. The data represent the mean ± standard deviation of three replicates. Means denoted by the same letters are not significantly different at *p* < 0.05. ND, not detected.

Cd exposure significantly induced PC biosynthesis in the roots of rice seedlings, with the highest accumulation observed under 1 mM S supplementation. PC2 levels were either undetectable or present only in trace amounts in root and leaf tissues of untreated control plants across all treatments, indicating tissue-specific and stress-responsive expression patterns. PC3 content peaked in Cd-stressed roots supplemented with 1 mM S, while control roots exhibited negligible levels. In leaves, PC3 remained relatively constant, regardless of Cd exposure. PC4 was exclusively detected in Cd-exposed root tissues, with significantly higher accumulation under 1 mM S supplementation. No measurable PC4 was observed in leaf tissues or control samples (Figure 5).

### 3.6. S Supplementation Altered Mineral Nutrient Composition in Rice Seedlings Under Cd Stress

S supplementation significantly affected the mineral nutrient composition of rice seedlings grown under Cd stress. Elemental profiling of root and shoot tissues after 15 days of hydroponic treatment with 0, 1, and 4 mM S revealed distinctive patterns of mineral nutrient accumulation. In shoot tissues, N, P, and K contents increased significantly with S supplementation. The highest shoot N content was observed at 4 mM S, while P content peaked at 1 mM S. K content increased steadily with S concentrations, reaching maximum values at both 1 and 4 mM S supplementation, suggesting improved nutrient uptake under Cd stress. In contrast, Ca and Mg concentrations in shoots decreased significantly with increasing S levels. However, root Mg content markedly increased at 4 mM S, indicating S-induced redistribution or localized accumulation. Shoot Ca content progressively decreased with increasing S concentration, while root Ca levels remained relatively unchanged.

S content significantly increased in both roots and shoots with S supplementation, particularly in roots at 4 mM S, confirming efficient uptake. Si levels remained consistently higher in roots than in shoots across all treatments, with minimal variation and no significant S-dependent effects. Micronutrients exhibited organ-specific responses. Fe and Cu contents slightly declined in shoot tissues as S concentration increased. Mn contents showed no notable variation. Among trace elements, Mo contents in shoots declined sharply at 4 mM S, representing the most pronounced reduction among all measured nutrients. B levels also decreased with S supplementation, while Zn content exhibited a slight, non-significant decline (Table 1).

### 3.7. Integrated Correlation Analysis

**Figure 6:**
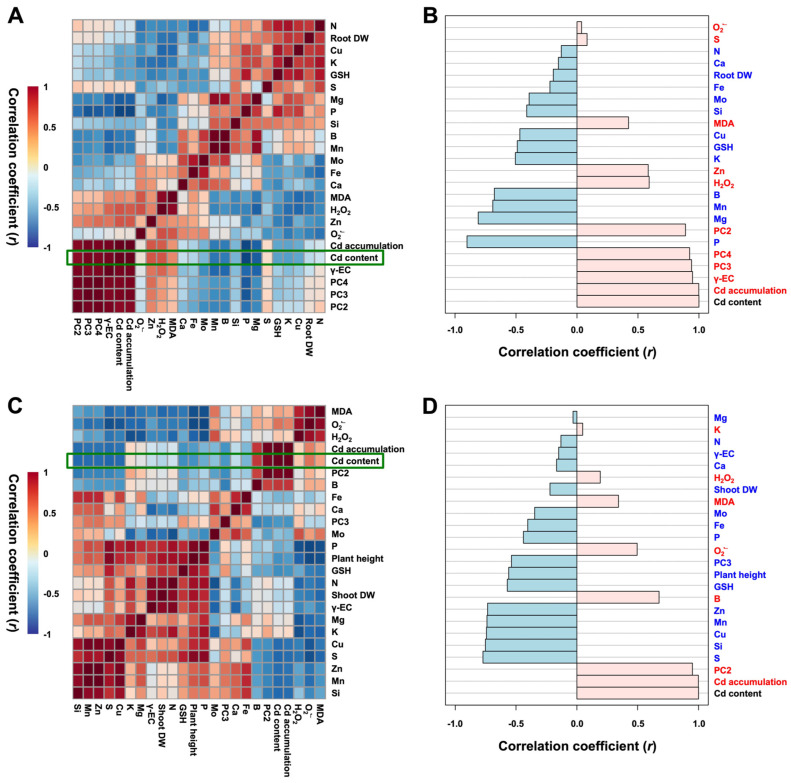
Heatmap responses of Pearson’s correlation coefficients (r) among measured variables, such as rice growth characteristics, thiol-based detoxification systems, mineral nutrient homeostasis, and oxidative stress, in roots (**A**,**B**) and shoots (**C**,**D**) of sulfur-supplemented rice seedlings exposed to cadmium stress. Positive and negative correlations are visualized using red and blue gradients, respectively.

Figure 6 presents heatmaps of Pearson correlation coefficient analysis among physiological and biochemical traits in rice roots (Figure 6A,B) and shoots (Figure 6C,D) under combined Cd and S treatments. In rice roots (Figure 6A), DW positively correlated with essential nutrients, such as N, P, K, and S, indicating their contribution to root biomass accumulation. It also correlated positively with GSH and negatively with oxidative stress markers (O_2_^•−^, H_2_O_2_, and MDA), suggesting that GSH-mediated reduction in oxidative stress supports root growth. In shoots (Figure 6C), plant height and shoot DW positively correlated with macronutrients (N, P, K, Mg, and S), micronutrients (Si, Mn, Cu, and Zn), and thiol-related metabolites (γ-EC and GSH), indicating that enhanced nutrient availability and antioxidant capacity contribute to the growth of aboveground tissues.

Comparative analysis of Cd-associated factors (indicated by green boxes) revealed that, in roots, Cd content showed a strong positive correlation with Cd accumulation, γ-EC, and PCs (PC2, PC3, PC4), and a negative correlation with GSH, indicating that roots consume GSH for PC synthesis to facilitate Cd chelation and sequestration into the vacuole (Figure 6B). In shoots, Cd content positively correlated with Cd accumulation, PC2, and oxidative stress markers and negatively correlated with GSH. These results suggest that Cd accumulation in shoots could be primarily mediated by PC2, with GSH being consumed in both PC synthesis and antioxidant defense responses (Figure 6D).

### 3.8. Integrated PCA Scores and Loading Plot Analysis

**Figure 7:**
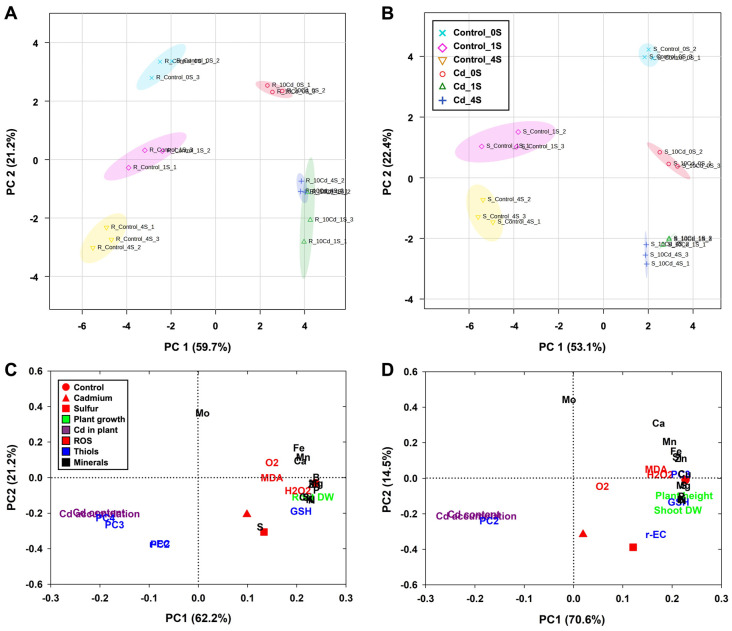
Principal component analysis (PCA) score (**A**,**B**) and loadings (**C**,**D**) plots of response variables, such as growth characteristics, thiol-based detoxification systems, mineral nutrient homeostasis, and oxidative stress, in roots (**A**,**C**) and shoots (**B**,**D**) sulfur−supplemented rice seedlings exposed to cadmium stress. Each data point represents the means of three replicates (*n* = 3).

PCA was performed to investigate the multivariate physiological and biochemical responses of rice seedlings subjected to Cd stress and S supplementation. The PCA score plots (Figure 7A,B) revealed distinct separations among treatment groups. PC1 accounted for 59.7% and 53.1% of the total variance in the root and shoot datasets, respectively, while PC2 accounted for 21.2% and 22.4%, respectively. Cd-treated groups clustered distinctly from the control groups along PC1, indicating strong Cd-induced alterations. Moreover, S supplementation in Cd-stressed plants shifted the distribution along PC2, with the effect being more pronounced in shoots than in roots.

Loading plots (Figure 7C,D) revealed that Cd-related traits, including Cd content and accumulation, were negatively associated with PC1 values in both roots and shoots. In roots, several PCs (PC2, PC3, and PC4) were also located on the negative side of PC1, whereas in shoots, only PC2 exhibited a similar pattern. Oxidative stress markers (O_2_^•−^, H_2_O_2_, and MDA) and thiol-related metabolites (γ-EC and GSH) contributed positively to PC1 in both tissues. However, these variables exhibited negative loadings along the PC2 axis. In contrast, mineral nutrients (N, P, K, and S) and growth traits (root and shoot DW and plant height) were positioned opposite to Cd-treated traits along PC1, suggesting that Cd stress impaired nutrient status and growth. Sulfur-treated samples were positioned between the control and Cd groups along PC1, indicating that S supplementation moderated physiological and biochemical alterations induced by Cd stress.

### 3.9. S-Driven Correlation Coefficient Network

**Figure 8:**
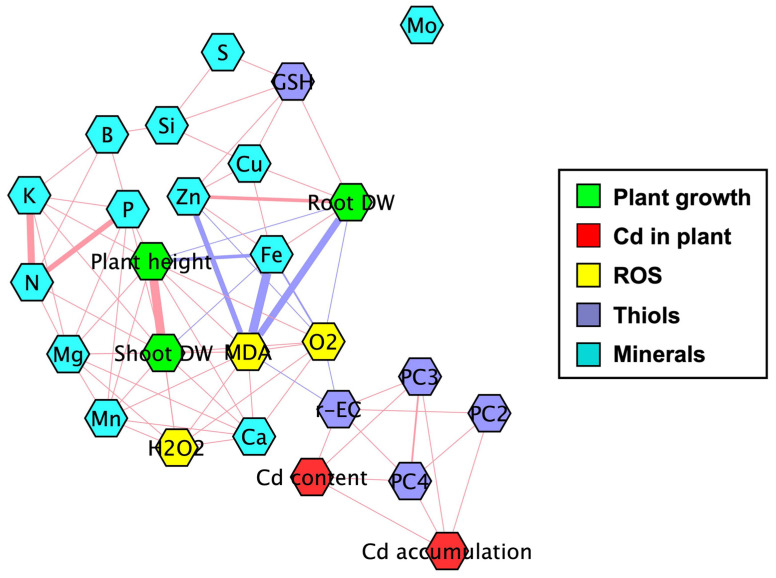
Pairwise correlation coefficient network (r ≥ |0.70|) constructed from the relative changes in response variables in roots and shoots of rice seedlings treated with sulfur under cadmium-stress or non-stress conditions. Red and blue colors denote positive and negative correlations between variables, respectively. The thickness of the correlation lines indicates the strength of the correlation coefficient (r) among variables.

A pairwise correlation coefficient network was constructed to elucidate the interrelationships among physiological and biochemical variables in rice seedlings subjected to Cd stress and S supplementation (Figure 8). In this network, each node represents a distinct variable, and edges denote significant correlations (*p* < 0.05). Red and blue edges indicate positive and negative correlations, respectively, with edge thickness reflecting the strength of the correlation. Under S treatment, particularly in Cd-stressed plants, notable positive correlations were observed among mineral nutrients (e.g., N, P, K, Ca, Mg, Mn, and Zn) and growth indicators, including shoot and root DW, and plant height. These correlations suggest that S supplementation enhances nutrient uptake and promotes biomass accumulation under stress conditions. Cd content and accumulation were directly and positively correlated with thiol-related metabolites, including γ-EC and PCs (PC2, PC3, and PC4), suggesting the activation of thiol-dependent detoxification pathways in response to Cd stress. In addition, these thiol-related metabolites were negatively correlated with oxidative damage indicators (MDA and O_2_^•−^) and root DW, suggesting a potential protective role of S-mediated thiol metabolism in mitigating Cd-induced oxidative stress (Figure 8).

## 4. Discussion

Cd is a highly toxic and persistent environmental contaminant, characterized by high bioavailability and phytotoxicity [27]. Due to its high solubility, Cd is readily absorbed by rice plants and translocated to aboveground tissues, where it disrupts critical cellular and physiological functions. This disruption inhibits plant growth and development, ultimately reducing rice yield and grain quality [28,29]. S-containing compounds have been shown to mitigate the effects of various abiotic stressors, including metal toxicity [30]. In this study, S supplementation alleviated Cd-induced growth inhibition in rice, with increasing S concentrations enhancing growth parameters under both Cd-stressed and control conditions. However, S supplementation under Cd stress also led to a significant increase in Cd accumulation in both plant roots and shoots. This observation is consistent with previous findings suggesting that S can enhance Cd accumulation in rice grains [31]. Although 4 mM S resulted in slightly lower Cd accumulation than 1 mM S, the difference was not significant, indicating that S promotes Cd uptake and translocation, while concurrently mitigating Cd-induced growth suppression.

Once internalized, Cd induces excessive production of ROS, including superoxide, H_2_O_2_, and hydroxyl radicals [32,33]. While ROS function as signaling molecules in plant stress-response pathways [34], their overproduction leads to cellular damage by targeting essential biomolecules, such as soluble carbohydrates, lipids, nucleic acids, and proteins, that are vital for processes, including photosynthesis, respiration, ion uptake, redox balance, and hormone regulation [35]. We observed that Cd stress significantly increased ROS levels in both rice roots and shoots and that elevated Cd uptake directly exacerbated oxidative stress, ultimately impairing plant growth. Cd accumulation is known to trigger excessive ROS production and MDA levels in plants [36]. Notably, S supplementation in Cd-stressed rice effectively reduced ROS levels in both tissues, highlighting the critical role of S in alleviating oxidative stress and mitigating the harmful effects of Cd toxicity.

Under Cd stress, plants activate antioxidant defense mechanisms to counteract Cd toxicity and improve tolerance [37]. In the present study, S treatments increased the levels of thiol-containing compounds, including γ-EC, GSH, and PCs, thereby reducing Cd-induced oxidative stress. S plays a key role in the biosynthesis of these ligands, which chelate Cd and facilitate its sequestration into vacuoles, reducing its toxic effects. These findings are consistent with those of previous studies [30,38]. Strong positive correlations between Cd content and both PCs and γ-EC, particularly in root tissues, further support the involvement of thiol-mediated Cd detoxification. S is a vital nutrient for the synthesis and regulation of γ-EC, GSH, and PCs, especially under Cd stress. Plants primarily absorb S as sulfate, which is transported to various tissues [39]. Once inside cells, sulfate is incorporated into Cys, the direct precursor for γ-EC, GSH, and PCs. The biosynthesis pathway begins with the formation of γ-EC from glutamate and cysteine, catalyzed by γ-EC synthetase [40]. Under Cd stress, increased pathway activity is required to meet the higher demand for GSH and PCs, as reflected by their elevated levels in this study [41]. GSH, synthesized from γ-EC and glycine by glutathione synthetase, serves multiple protective roles [42]. It not only directly neutralizes ROS but also participates in the regeneration of other antioxidants via the ascorbate–glutathione cycle. Additionally, GSH serves as a substrate for PC synthesis, catalyzed by PC synthase [43], thereby contributing to the chelation and sequestration of Cd for stress mitigation.

In this study, increased Cd accumulation in rice roots was accompanied by elevated levels of thiol-containing compounds, particularly γ-EC and PCs, suggesting that a substantial portion of Cd is chelated by thiol-containing peptides in the roots and sequestered into vacuoles, thereby restricting its translocation to the shoots, as shown previously [30]. Although γ-EC levels increased under Cd stress, GSH levels declined markedly in both roots and leaves, indicating a metabolic shift during Cd detoxification in which GSH is rapidly consumed—either directly for ROS neutralization or as a precursor for PC synthesis—while γ-EC is primarily channeled toward PC production. Tissue-specific biochemical responses further revealed that roots, as the primary site of Cd uptake, exhibited high Cd concentrations and robust defense activation, with the accumulation of γ-EC, GSH, and PCs in root tissues suppressing oxidative stress, reducing cytosolic Cd toxicity, and limiting Cd transport to aboveground parts. In contrast, leaves exhibited weaker induction of thiol-based defenses, along with lower sulfur transport and assimilation compared to roots (Table 1). Consequently, although Cd accumulation in leaves was lower than in roots, the reduced sulfur availability may limit the synthesis of S-containing thiol compounds, leading to more pronounced oxidative damage in leaves. This may also reflect a delayed or insufficient activation of defense pathways in leaves, particularly within the short 15-day exposure period. Moreover, leaf tissues are inherently more vulnerable to oxidative stress due to high redox activity and limited detoxification capacity. Therefore, investigating long-term responses will be important for fully elucidating these adaptive mechanisms. Supporting this, PCA loading plots revealed clear treatment separation and strong loadings of thiol-related variables in roots. In contrast, shoot tissues exhibited lower levels of these compounds, indicating more effective detoxification in belowground tissues.

Furthermore, S supplementation significantly altered the mineral nutrient composition of rice plants under Cd stress, consistent with previous findings [44,45,46]. Notably, N, P, and K levels increased in both roots and shoots, indicating improved macronutrient uptake likely associated with enhanced root metabolic activity. Similar increases in P and K following S treatment have been reported in earlier studies [47,48]. These macronutrients play essential roles in protein synthesis, energy production, and osmotic regulation, collectively contributing to stress resistance and plant growth. Multivariate analysis revealed positive correlations between macronutrients (N, P, and K) and various micronutrients and growth traits, except for Mo. Conversely, Ca and Mg levels declined, potentially due to competitive ion uptake or antagonistic interactions with sulfate [49]. The increased absorption of S itself indicates active uptake by rice plants and its utilization in amino acid and protein synthesis [50]. Si levels increased in roots but decreased in leaves, suggesting limited root-to-shoot translocation under S-enriched conditions. Micronutrient profiles also shifted: Fe, Mn, and Mo levels decreased in both tissues, while Cu levels increased. Previous studies have similarly reported reduced Fe levels in S-supplemented rice shoots [51,52]. Zn and B levels declined in roots; however, B increased in shoots, indicating possible upward movement.

## 5. Conclusions

This study demonstrated that S supplementation significantly enhanced Cd tolerance in rice (*Oryza sativa* L. cv. Shindongjin) by mediating a series of protective physiological and biochemical responses. S application activated antioxidant defense systems, promoted thiol-mediated detoxification pathways, and stabilized mineral nutrient homeostasis, thereby alleviating oxidative stress and lipid peroxidation induced by Cd exposure. Notably, S treatment markedly upregulated the biosynthesis of γ-EC, GSH, and PCs, particularly in root tissues, suggesting a spatially regulated detoxification response at the primary site of Cd uptake. Although S supplementation increased Cd accumulation in plant tissues, it also induced biochemical adjustments that enhanced physiological resilience and optimized the uptake and distribution of macro- and micronutrients under Cd stress, thereby improving nutritional status and facilitating stress recovery. Collectively, these findings highlight the multifaceted role of S in improving Cd tolerance in rice. Furthermore, since the current study findings are based on hydroponic experiments, further soil-based and field studies are necessary to evaluate S-mediated Cd detoxification in rice under a complex soil environment.

## Figures and Tables

**Table 1:** Effect of sulfur (S) on macro- and micronutrient contents in roots and shoots of rice seedlings grown in cadmium (Cd)-treated hydroponics. The data represent the mean ± standard deviation of three replicates. Means within a column followed by the same letters are not significantly different at *p* < 0.05. DW, dry weight.

Plant Part	Cd (µM)	S (mM)	Macronutrient (g kg^−1^ DW)						Micronutrient (mg kg^−1^ DW)						
			N	P	K	Ca	Mg	S	Si	Fe	Mn	Cu	Zn	B	Mo
Root	Control	0	22.9 c	10.4 b	2.63 bc	0.79 a	2.03 a	0.54 c	126 ab	993 a	35 a	94 c	328 ab	42 a	1236 a
		1	26.3 b	11.8 a	2.83 c	0.58 bc	1.84 b	1.49 c	103 bc	719 b	21 b	171 a	297 bc	31 c	85 c
		4	28.2 a	12.2 a	3.72 a	0.42 c	1.83 b	25.10 a	144 a	600 b	19 b	142 b	264 c	33 b	64 c
	10	0	21.3 d	9.8 b	1.72 d	0.62 ab	1.39 d	0.45 c	88 c	1059 a	12 c	38 d	334 ab	29 cd	818 b
		1	24.9 b	8.1 c	2.43 bc	0.54 bc	1.51 c	1.41 c	94 bc	627 b	12 c	92 c	362 a	28 d	55 c
		4	25.2 b	8.5 c	2.13 cd	0.57 bc	1.32 d	20.90 b	108 bc	702 b	11 c	86 c	337 ab	27 d	46 c
Shoot	Control	0	34.7 e	16.2 c	4.70 b	3.50 a	4.37 ab	2.48 c	110 a	445 a	575 a	23 a	85 a	30 abc	920 b
		1	38.5 b	20.9 a	5.10 a	2.46 b	4.41 a	3.12 a	88 b	312 bc	453 b	23 a	77 b	27 c	24 d
		4	40.0 a	19.1 b	5.00 a	1.68 d	3.96 c	2.81 b	79 bc	288 bc	282 c	20 b	60 c	31 abc	11 e
	10	0	33.3 f	9.9 e	3.65 c	2.57 b	3.21 d	1.02 e	59 cd	294 bc	113 d	15 d	49 d	30 bc	941 a
		1	36.5 d	14.7 d	4.77 b	2.58 b	4.13 bc	1.25 d	52 d	273 c	125 d	16 d	49 d	34 a	32 c
		4	37.2 c	14.7 d	5.10 a	1.96 c	4.18 ab	1.23 d	45 d	320 b	108 d	17 c	47 d	33 ab	17 e

## Data Availability

Data are contained within this article.

## Abbreviations

The following abbreviations are used in this manuscript:

ANOVA	Analysis of variance
As(III)	Arsenite
DW	Dry weight
GSH	Glutathione
H_2_O_2_	Hydrogen peroxide
MDA	Malondialdehyde
PC	Phytochelatin
PCA	Principal component analysis
ROS	reactive oxygen species
SD	standard deviations
SO_4_^2−^	sulfate
O_2_^•−^	superoxide anion radicals
TBARS	thiobarbituric acid-reactive substances
γ-EC	γ-glutamylcysteine
